# Supplementary material for: Stacking Model‐Based Classifiers for Dealing With Multiple Sets of Noisy Labels
Source: Biom J. 2025 Mar 12;67(2):e70042. doi: 10.1002/bimj.70042 (PMC11898607; doi:10.1002/bimj.70042)
Supplement: Supplementary file 1 — Supporting Information [file BIMJ-67-e70042-s001.zip › density-based-ensemble-model-main/competitors_real_data/netlab-master/netlab-master/help/mdn.htm]

Netlab Reference Manual mdn


# mdn

## Purpose

Creates a Mixture Density Network with specified architecture.

## Synopsis

```
net = mdn(nin, nhidden, ncentres, dimtarget)
net = mdn(nin, nhidden, ncentres, dimtarget, mixtype, ...
	prior, beta)
```

## Description

`net = mdn(nin, nhidden, ncentres, dimtarget)` takes the number of
inputs,
hidden units for a 2-layer feed-forward
network and the number of centres and target dimension for the
mixture model whose parameters are set from the outputs of the neural network.
The fifth argument `mixtype` is used to define the type of mixture
model. (Currently there is only one type supported: a mixture of Gaussians with
a single covariance parameter for each component.) For this model,
the mixture coefficients are computed from a group of softmax outputs,
the centres are equal to a group of linear outputs, and the variances are
obtained by applying the exponential function to a third group of outputs.

The network is initialised by a call to `mlp`, and the arguments
`prior`, and `beta` have the same role as for that function.
Weight initialisation uses the Matlab function `randn`
and so the seed for the random weight initialization can be
set using `randn('state', s)` where `s` is the seed value.
A specialised data structure (rather than `gmm`)
is used for the mixture model outputs to improve
the efficiency of error and gradient calculations in network training.
The fields are described in `mdnfwd` where they are set up.

The fields in `net` are

```
  type = 'mdn'
  nin = number of input variables
  nout = dimension of target space (not number of network outputs)
  nwts = total number of weights and biases
  mdnmixes = data structure for mixture model output
  mlp = data structure for MLP network
```

## Example

```
net = mdn(2, 4, 3, 1, 'spherical');
```

This creates a Mixture Density Network with 2 inputs and 4 hidden units.
The mixture model has 3 components and the target space has dimension 1.

## See Also

`mdnfwd`, `mdnerr`, `mdn2gmm`, `mdngrad`, `mdnpak`, `mdnunpak`, `mlp`

---

**Pages:**
Index


---

Copyright (c) Ian T Nabney (1996-9)

David J Evans (1998)
